# Phenotypic characterization and validation of provitamin A functional genes in early maturing provitamin A‐quality protein maize (*Zea mays*) inbred lines

**DOI:** 10.1111/pbr.12798

**Published:** 2019-12-20

**Authors:** Ebenezer Obeng‐Bio, Baffour Badu‐Apraku, Beatrice Elorhor Ifie, Agyemang Danquah, Essie T. Blay, Mustapha Abu Dadzie

**Affiliations:** ^1^ West Africa Centre for Crop Improvement (WACCI) University of Ghana Accra Ghana; ^2^ CSIR‐Crops Research Institute Kumasi Ghana; ^3^ International Institute of Tropical Agriculture (IITA) Ibadan Nigeria; ^4^ Cocoa Research Institute of Ghana New Tafo‐Akim Ghana

**Keywords:** candidate genes, early maturing maize, inbred lines, provitamin A

## Abstract

The number of drought and low‐N tolerant hybrids with elevated levels of provitamin A (PVA) in sub‐Saharan Africa could increase when PVA genes are optimized and validated for developed drought and low‐N tolerant inbred lines. This study aimed to (a) determine the levels of drought and low‐N tolerance, and PVA concentrations in early maturing PVA‐quality protein maize (QPM) inbred lines, and (b) identify lines harbouring the *crtRB1* and *LcyE* genes as sources of favourable alleles of PVA. Seventy early maturing PVA‐QPM inbreds were evaluated under drought, low‐N and optimal environments in Nigeria for two years. The inbreds were assayed for PVA levels and the presence of PVA genes using allele‐specific PCR markers. Moderate range of PVA contents was observed for the inbreds. Nonetheless, TZEIORQ 55 combined high PVA concentration with drought and low‐N tolerance. The *crtRB1‐3′TE* primer and the KASP SNP (snpZM0015) consistently identified nine inbreds including TZEIORQ 55 harbouring the favourable alleles of the *crtRB1* gene. These inbreds could serve as donor parents of the favourable *crtRB1‐3′TE* allele for PVA breeding in maize.

## 1 INTRODUCTION

Maize production in sub‐Saharan Africa (SSA) is limited by drought and low‐N which are the two most frequent yield reduction factors in the sub‐region (Badu‐Apraku, Fakorede, Oyekunle, & Akinwale, 2011). The two stresses significantly contribute to low grain yield in SSA annually hovering around 1.8 t/ha (FAOSTAT, 2016). However, studies have revealed that genetic improvement in maize for stress tolerance could result in genetic gains under drought (Badu‐Apraku et al., 2011; Edmeades, Bolaños, Chapman, Lafitte, & Bänziger, 1999) and under low‐N (Badu‐Apraku, Oyekunle, Akinwale, & Aderounmu, 2013).

Vitamin A is an essential micro‐nutrient required by human beings for improved eye sight and enhanced immune system. However, humans must consume either vitamin A or provitamin A (PVA) carotenoids, from which we synthesize vitamin A. The PVA maize has the potential of supplying more than 15.0 µg/g dry weight (DW) PVA which is the set target to combat vitamin A deficiency (VAD) especially among children and women when common amounts of PVA maize are consumed compared to just about 2.0 µg/g DW found in the commonly cultivated and consumed yellow maize (HarvestPlus, 2004; Pixley et al., 2013; Simpungwe et al., 2017).

Several studies (Li, Tayie, Young, Rocheford, & White, 2007; Mugode et al., 2014; Muzhingi et al., 2011) were conducted on the retention of PVA carotenoids and their bioavailability when consumed. This was necessary because the traditional African household processing methods could result in PVA reduction of about 20%–30%. The conversion factor of yellow maize β‐carotene to retinol (the form of vitamin A used by the body) by weight was 3.2 ± 1.5 to 1 (Muzhingi et al., 2011). Preliminary PVA target density of 15 µg/g DW (HarvestPlus, 2004) was, therefore, set to provide an estimated 50% dietary requirement of 275 µg of retinol in children and about 500 µg in women based on common quantities of PVA maize consumed (Simpungwe et al., 2017). For example, with 15 µg/g DW PVA which translates into about 5 µg of retinol (using a conversion factor of 3:1), a child could meet the estimated 50% daily retinol requirement of 275 µg by consuming about 825 µg DW PVA obtainable from an average quantity of 55 g of PVA maize consumed. In view of this potential of PVA maize, there has been some progress with respect to the improvement of PVA carotenoids in developed maize varieties in SSA, with over 40 PVA varieties released (Andersson, Saltzman, Virk, & Pfeiffer, 2017; Listman et al., 2019). However, the PVA concentrations of these varieties range between 6 and 10 µg/g (Andersson et al., 2017). Thus, much could be done regarding the number of hybrids released in SSA and the levels of PVA concentrations. This calls for the need for detection and validation of the candidate genes especially phytoene synthase1 (*PSY1*), lycopene epsilon cyclase (*LcyE*) and β‐carotene hydroxylase1 (*crtRB1*) which have been identified to regulate the key steps involved in the accumulation of PVA carotenoids in maize endosperm (Wurtzel, Cuttriss, & Vallabhaneni, 2012). Among these three genes, *crtRB1* (with the alleles *crtRB1*‐*5′TE and crtRB1‐3′TE*) is the most favourable for increased β‐carotene levels (Babu, Rojas, Gao, Yan, & Pixley, 2013). Also, two of the three significant polymorphic alleles of *LcyE*, that is *LcyE*‐5′TE and *LcyE*‐3*′* indel, have been validated using 26 different tropical segregating populations (Babu et al., 2013).

The quality protein maize (QPM) has about twice of its lysine and tryptophan contents compared to the conventional maize. In whole grain per sample, a maize genotype is classified as QPM when the tryptophan content exceeds 0.075% (Teklewold et al., 2015; Vivek, Krivanek, Palacios‐Rojas, Twumasi‐Afriyie, & Diallo, 2008). The development of QPM was successful due to (a) the presence of the recessive opaque 2 alleles (*o2o2*) which are lacking in the conventional maize counterparts, (b) enhancers of the endosperm containing the *o2*‐gene for increased levels of tryptophan and lysine and (c) modifying genes responsible for the hardness of the *o2*‐induced soft endosperm (Twumasi‐Afriyie et al., 2016). In addition, the Kompetitive Allele‐Specific PCR (KASP) SNP, snpZM0015 located inside the *crtRB1* gene on chromosome 10, has been optimized and recommended for accelerating PVA improvement in maize (Intertek Group Plc., Sweden, unpublished).

The early maturing maize inbred lines with backgrounds of PVA, as well as essential amino acids, that is tryptophan and lysine that qualify maize genotypes as QPM (Atlin et al., 2011; Krivanek, Groote, Gunaratna, Diallo, & Friese, 2007), are a new set of inbred lines developed by the Maize Improvement Programme (MIP) at the International Institute of Tropical Agriculture (IITA), Ibadan‐Nigeria (Badu‐Apraku & Fakorede, 2017). The lines were selected based on the deep orange colour (colour scores > 8.0) of the kernels (Chandler et al., 2013) which made them distinct from the typical yellow cultivars. This implied that the inbred lines at least had elevated levels of PVA compared to the typical yellow maize which usually have PVA levels ranging from 0.5 µg/g to 1.5 µg/g DW (Egesel, Wong, Lambert, & Rocheford, 2003). Studies by Sivaranjani, Prasanna, Hossain, and Santha, (2013) have revealed positive correlation between kernel colour and total carotenoid concentration. However, a genotype can have higher levels of total carotenoids with lower concentrations of PVA. Azmach, Gedil, Menkir, and Spillane, (2013) have found weak or zero correlations between kernel colour and provitamin A carotenoids. Therefore, orange colour per se is not reliable in determining PVA levels of maize genotypes (Azmach et al., 2013; Menkir, Liu, White, Maziya‐Dixon, & Rocheford, 2008; Menkir & Maziya‐Dixon, 2004). Although kernel colour was initially used to select inbred lines purporting to harbour elevated levels of PVA, in the present study, chemical analysis was used to determine the levels of these carotenoids. In addition, a clear knowledge of the presence of the PVA favourable genes to accelerate PVA accumulation in the inbreds is needed to guide breeding strategies for developing superior early PVA‐QPM hybrids. This study was, therefore, conducted to (a) determine the levels of drought and low‐N tolerance, and PVA concentration in the early PVA‐QPM inbred lines, and (b) identify lines harbouring the *crtRB1* and *LcyE* genes as sources of favourable PVA alleles to serve as donor parents.

## 2 MATERIALS AND METHODS

### 2.1 Plant materials

Seventy early maturing PVA‐QPM inbreds of maize (*Zea mays* L.) including six checks developed in the IITA‐MIP were used in the present study. The inbred lines were extracted from the variety 2009 TZE‐OR2 DT STR QPM that was developed for multiple stress (drought, low‐N and *Striga*) tolerance and elevated concentrations of PVA, tryptophan and lysine. The crosses that led to the development of the source open‐pollinated variety from 2007 to 2010 and extraction of S_1_ to S_7_ inbred lines from 2011 to 2015 vis‐à‐vis screening under drought and *Striga* environments have been previously described (Badu‐Apraku & Fakorede, 2017).

At the end of the 2015 growing season, 150 S_7_ inbred lines with varying reactions to the multiple stresses were analysed for tryptophan content in the IITA chemical laboratory in Ibadan, Nigeria, and 73 (only those with >0.075%) were kept for further use (Badu‐Apraku & Fakorede, 2017). From the 73 early PVA‐QPM inbreds, 64 plus six checks were selected for the present study.

### 2.2 Field trials

Evaluation of the 70 inbred lines was carried out under drought, low‐N and optimal conditions in Nigeria. The drought experiments were conducted at Ikenne (6°50′N, 30°45′E, 62 m altitude, 1,200 mm mean rainfall annually) in the 2016/2017 and 2017/2018 dry seasons. Drought stress was achieved by supplying 17 mm of sprinkler irrigation water in a week up to 25 days after planting (DAP) after which the irrigation was terminated and the maize plants depended on the available soil moisture to reach physiological maturity. The managed drought trials received NPK fertilizer at the rate of 60 kg/ha each of N, P and K (15–15–15) during planting. Additionally, top dressing was done with 60 kg/ha of N (supplied as urea) at 3 weeks after planting (WAP).

Evaluation of the inbreds under low‐N (30 kg/ha) conditions was carried out at Ile‐Ife (7°30′N, 5°31′E, and 240 m altitude, 1,250 mm mean rainfall annually) and Mokwa‐(10°20′N, 5°6′E, 459 m above sea level, 1,050 mm mean rainfall annually) in the 2016 and 2017 major growing seasons. Low soil nitrogen conditions at both locations were accomplished by depleting the fields of N through continuous cultivation of densely populated maize without fertilizer application for three cropping seasons and complete removal of crop residues at the end of every harvest. Prior to field preparation, topsoil samples were collected at the depth of 0–15 cm for analysis of the contents of nitrogen (N), phosphorus (P) and potassium (K) using the Kjeldahl digestion and colorimetric procedure (Bremner & Mulvaney, 1982) at the IITA analytical services laboratory, Ibadan, Nigeria. The low‐N experimental field at Mokwa had 0.085 g/kg N, 6.32 g/kg P and 0.20 g/kg K, whereas that of Ile‐Ife contained 0.084 g/kg N, 2.05 g/kg P and 0.358 g/kg K. Based on the soil tests, NPK fertilizer was formulated using urea (N source), triple superphosphate (P_2_O_5_ source) and muriate of potash (K_2_O source), respectively, and it was applied immediately after thinning (2 WAP). The urea provided a basal available N of 15 kg/ha, P_2_O_5_ and K_2_O fertilizers supplied 60 kg/ha each of P and K. Additionally, top dressing of 15 kg/ha of N (supplied as urea) was done at 4 WAP to bring the total available N received on the low‐N fields to 30 kg/ha.

The inbreds were planted under optimal growing conditions at Ikenne, Ile‐Ife and Mokwa in the 2016 and 2017 rainy seasons. The optimal trials received NPK (15:15:15) fertilizer at 60 kg/ha each for N, P and K at 2 WAP immediately after thinning. Top dressing was done at 4 WAP with additional 30 kg N/ha. In all the experiments, 7 × 10 randomized incomplete‐block design with two replications was used. The experimental units consisted of 3‐m‐long one‐row plots with a spacing of 0.75 × 0.40 m. Three seeds were sown per hill and later, seedlings were thinned to two at 2 WAP to give a population density of about 66,667 plants/ha. Weeds were managed in all trials by the application of pre‐ and postemergence herbicides, atrazine (Primextra) and gramoxone (Paraquat), respectively, each at the rate of 5 L/ha.

### 2.3 Agronomic data collection

Based on individual plots, data were taken for 50% days to anthesis (DA) and silking (DS) as well as plant and ear heights (PLHT and EHT). Plant and ear aspects (PASP and EASP) were rated on a scale of 1–9 (1 = excellent plants or ears and 9 = extremely poor plants or ears). The difference between DA and DS was calculated as anthesis–silking interval (ASI). The number of ears per plant (EPP) was obtained as the ratio of the number of harvested ears in a plot to the total number of plants in that plot. At 70 DAP, visual ratings for stay‐green characteristic (STGR) were carried out for the trials under drought and low‐N using a scale of 1–9 (1 = less than 10% dead leaf area and 9 = more than 80% dead leaf area). The harvested ears from each plot under the two stress conditions were shelled to measure grain weight. Grain moisture content was determined using Kett moisture tester PM‐450. Grain weight was adjusted to 15% moisture content, and grain yield (GY) in kg/ha was computed on plot basis. For the optimal trials on the other hand, an assumption of 80% shelling percentage was considered per plot to compute GY from ear weight adjusted to 15% moisture content.

### 2.4 Production of kernel samples for carotenoid analysis

The inbred lines were planted under well‐watered growing conditions at Mokwa (9°18′N, 5°4′E, altitude 457 m) and IITA Ibadan (7°28′11.99″N, 3° 53′2.88″E, altitude 190 m) in 2018 to produce kernel samples for carotenoid analysis. Four‐metre‐long single‐row plots with two replications and each with inter‐row and intra‐row spacing of 0.75 and 0.40 m, respectively, were used in each location. Seed samples were produced by controlled self‐pollination (S_8_) of all plants in each plot. Ears of self‐pollinated lines were harvested, well‐dried under ambient temperature with minimal exposure to direct sun light and shelled separately. For each inbred line in a location, samples of 100 kernels were drawn by taking equal kernels from ears within a plot to represent the genotype. The samples were sent to the IITA nutritional laboratory for carotenoids and tryptophan analyses.

### 2.5 Analysis of provitamin A carotenoids

Carotenoids were extracted and quantified by HPLC at the IITA nutritional laboratory, Ibadan, Nigeria. The protocol for extraction and carotenoid analysis was based on the procedure described in Howe and Tanumihardjo (2006). Total carotenoids were computed as the sum of concentrations of α‐carotene, β‐carotene, lutein, zeaxanthin and β‐cryptoxanthin. PVA was computed as the sum of β‐carotene, and half of each of β‐cryptoxanthin and α‐carotene contents, since β‐cryptoxanthin and α‐carotene contribute half (50%) of the value of β‐carotene as PVA (US Institute of Medicine, 2001). The selected early maturing PVA‐QPM inbred lines were analysed for only tryptophan content but not lysine or both in whole grain flour. This is because lysine content of the maize endosperm is highly correlated with that of tryptophan (greater than 0.9; Nurit, Tiessen, Pixley, & Palacios‐Rojas, 2009; Villegas, Vasal, & Bjarnason, 1992). Moreover, analysis for tryptophan is far cheaper than lysine and so it is economically prudent for the breeder to use tryptophan content to determine the nutritional potential of QPM genotypes at early breeding stages (Nurit et al., 2009; Villegas et al., 1992). Tryptophan was quantified by the colorimetric method (Herbabdes & Bates, 1969). Values of all carotenoids and tryptophan for each sample were obtained from two technical replications from each field replication to increase accuracy in the carotenoid quantification.

### 2.6 Leaf sample collection and DNA extraction

Seventy (64 PVA‐QPM plus 6 checks) inbred lines were planted at IITA, Ibadan (7°28′11.99″N, 3°53′2.88″E, altitude 190 m) in 2018 to produce leaf samples for DNA extraction. Ten plants were planted per inbred line at a spacing of 0.75 × 0.20 m, and leaf samples were collected 2 WAP. Leaves from the 10 plants (one leaf per plant) were bulked and freeze‐dried to represent an inbred line. Genomic DNA samples were isolated from the freeze‐dried leaf tissues of each inbred following the DArT DNA extraction protocol (https://ordering.diversityarrays.com/files/DArTDNAisolation.pdf). The DNA concentration was obtained by spectrometry measurement using NanoDrop 8000 machine (Thermo Scientific), and DNA quality was confirmed by running DNA samples on 0.8% agarose gel. Short or degraded DNA was eliminated, and DNA concentrations of 30 ng/μl were used.

### 2.7 PCR‐based genotyping

PCR‐based functional markers of the *crtRB1* and *LcyE* genes were tested across the 70 inbred lines. Primer sequence, PCR conditions and thermal cycling profiles were modified based on the method of Yan, Kandianis, Harjes, and Bai (2010) and Harjes et al. (2008). The primer pair, forward: crtRB1_3TE_T_F1‐TCTTTTCACCGCCCTTTT; and reverse: crtRB1_3TE_T_R1c –AACAGCAATACAGGGGAC, used to amplify the crtRB1‐3′TE marker and primer pair, forward: –CGTGACCATATGTACACTCTC; reverse: ‐TCACCGGATATGGCACTGG, used to amplify the LcyE_5′TE were ordered from Integrated DNA Technology Inc (IDT). PCR amplifications of 1 µl sample DNA (30 ng/µl) were performed using OneTaq Quick‐Load 2× Master Mix with Standard Buffer (New England Biolabs Inc.) with a mixture composed of 5 µl of 2× Master Mix with Standard Buffer, 0.5 µl of each primer and ultra‐pure water making up to 10 µl total reaction volume. PCR thermal cycling profile was 1 cycle of initial denaturation at 94°C for 3 min, followed by 35 cycles of denaturation at 94°C for 30 s, annealing at 58°C for 1 min and extension at 68°C for 1 min. This was followed by 1 cycle of final extension at 68°C for 5 min and hold at 4°C. Fragments in the PCR products were resolved on 2% agarose gel. The polymorphic sites of the crtRB1‐3′TE gene used have a 325/1,250 bp indel, with 595 bp amplicon being the favourable allele, while 920 and 1,845 bp are the unfavourable alleles. Also, the polymorphic sites of the LcyE‐5′TE gene used have a 401/1,567 bp indel, with 595 bp amplicon being the favourable allele and 1,845 bp being the unfavourable allele (Azmach et al., 2013).

### 2.8 Kompetitive allele‐specific PCR (KASP) genotyping

Genomic DNA isolated from leaf tissue of the 70 maize inbred lines was used as template for the KASP genotyping reaction. Sample DNA was diluted to a working concentration of 30 ng/µl for use in the KASP genotyping reaction. KASP assay, snpZM0015, was used to investigate the presence and/or absence of the favourable alleles for the crtRB1 gene. KASP reaction was performed in a 96‐well plate in a reaction volume of 10 µl consisting 5 µl template DNA and 5 µl of the prepared genotyping mix (2× KASP master mix and primer mix). Protocols for the preparation and running of KASP reactions are presented in the KASP manual (http://www.kbioscience.co.uk, accessed on 2nd July 2018). KASP assay kit was purchased from LGC Genomics (LGC Group). All amplification reactions were performed using the Roche LightCycler 480 II (LC480 II) System (Roche Life Science) at the Bioscience Centre of IITA Ibadan, Nigeria. Amplification condition was as follows: 1 cycle of KASP special *Taq* activation at 94°C for 15 min, followed by 36 cycles of denaturation at 94°C for 20 s, and annealing and elongation at 60°C (dropping 0.6°C per cycle) for 1 min. Endpoint detection of the fluorescence signal was acquired for 1 min at 30°C using the same instrument. Genotyping result was analysed using KlusterCaller software (LGC Group), and genotyping data were visualized as cluster plots and downloaded using SNPviewer software (LGC Group). Allele calls for the SNP were made based on validation result provided by Intertek (Intertek Group Plc.), as homozygous AA for favourable allele, homozygous GG for unfavourable allele or heterozygous AG for both alleles.

### 2.9 Statistical analysis

Agronomic data recorded for the inbreds were subjected to analysis of variance (ANOVA) under each and across environments using PROC GLM in Statistical Analysis Software (SAS) version 9.4 with a random statement and a test option (SAS Institute, 2012), and means were separated using standard error of difference (SED). In the ANOVA, location by year combination was considered as an environment. Environments, replications within environments and incomplete blocks within replications environment interactions were treated as random factors, while inbred was regarded as a fixed factor. PVA carotenoid data were transformed using natural logarithm as the ratios were not expected to follow a normal distribution curve. ANOVA was also performed for PVA carotenoids for each and across the two locations (Mokwa and Ibadan). Mean concentrations of PVA and the component carotenoids measured for Ibadan and Mokwa were compared by performing a two‐tailed independent samples *t* test with equal pooled variance using SAS. Furthermore, effect size of the significant *t*‐values was determined by the estimated Cohen's *d* (Cohen, 1988) as follows:

$$d=t\sqrt{\frac{N1+N2}{N1N2}}$$

where *t* = *t*‐value, and *N*1 and *N*2 = number of observations in samples one and two, respectively. A measure of effect size (Cohen's *d* estimates) up to 0.20, 0.50 and 0.80 was classified as small, medium and large, respectively. Repeatability of the traits was calculated on mean basis using the following formula:

$$R=\frac{\sigma_{G}^{2}}{\sigma_{G\;}^{2}+\frac{\sigma_{\text{GE}}^{2}}{e}+\frac{\sigma^{2}}{\mathrm{re}}}$$

where $\sigma_{G}^{2}$ is the genotypic variance, $\sigma_{\text{GE}}^{2}$ is genotype × environment variance, $\sigma^{2}$ is error variance, *e* is number of environments, and *r* is number of replications. Variances were estimated using REML method in SAS MIXED procedure.

## 3 RESULTS

### 3.1 Analysis of variance for agronomic traits and provitamin A carotenoids

ANOVA under two drought environments revealed significant (*p < *.01) variation among environments (E) and inbreds (G) except ASI, PASP, EASP and EPP for environments (Table 1). However, inbred × environment interactions (GEI) were significant (*p* < .05) for only GY and ASI. Across the three low‐N environments, significant (*p < *.01) variations were observed among E, G and GEI mean squares for the traits measured except GEI mean squares for EHT. High repeatability (*R*) values were estimated for PASP, EASP, EPP and STGR compared with that for GY (56%) under drought. Under low‐N, GY had a relatively low R estimate compared with the moderately high values estimated for DA, DS, PLHT and EHT. Under optimal environments, significant (*p* < .05) differences were found among E, G and GEI mean squares for all traits. The few exceptions were GEI mean squares for DA, PLHT and EASP (Table 2). Across the eight test environments, there were significant (*p < *.01) differences among E, G, research condition and GEI mean squares for the agronomic traits except GEI mean squares for DA. Significant (*p < *.01) *G* × research condition mean squares were detected for ASI, EASP and STGR. Relatively high R estimate of 58% was observed for GY under optimal conditions compared to the estimates under drought and low‐N conditions.

**Table 1:** Mean squares and heritability estimates of 70 early maturing provitamin A‐quality protein maize inbred lines evaluated under drought at Ikenne in the 2016/2017 and 2017/2018 dry seasons and under low‐N conditions at Ile‐Ife and Mokwa in the 2016 and 2017 growing seasons

Source	*df*	GY	DA	DS	ASI	PLHT	EHT	PASP	EASP	EPP	STGR
Induced drought condition											
Env	1	480,952.72**	278.01**	284.01**	0.03	19,881.61**	9,165.73**	2.41	0.01	0.07	38.63**
Rep(Env)	2	11,038.47	2.8	3.21	11.51*	294.78*	66.58	0.46	2.35	0.03	5.16**
Block(Env × Rep)	36	65,311.25**	17.35**	21.68**	3.93	330.43**	109.03**	1.92**	3.60**	0.06**	1.71**
Inbred	69	131,623.07**	14.85**	21.2**	5.92**	475.15**	165.85**	1.86**	4.29**	0.08**	2.33**
Env × Inbred	69	55,852.2*	3.9	7.15	4.96*	91.73	59.43	0.64	1.11	0.02	0.92
Error	102	34,510.18	6.81	10.62	3.45	93	54.13	0.62	1.07	0.03	0.64
Repeatability (R)	—	0.56	0.65	0.61	0.18	0.83	0.65	0.70	0.77	0.75	0.65
Low‐N condition											
Env	2	33,597,047.68**	41.40**	318.44**	115.78**	6,370.30**	474.82**	56.12**	24.99**	4.35**	170.53**
Rep(Env)	3	673,976.31**	4.03	3.47	0.05	1,170.16**	946.18**	3.13**	3.25	0.06	0.37
Block(Env × Rep)	54	302,824.7**	2.15	2.54	0.96	277.44**	108.01*	1.61**	2.60**	0.04*	0.72*
Inbred	69	526,260.25**	9.38**	13.03**	4.75**	749.38**	186.99**	1.28**	3.18**	0.11**	1.57**
Env × Inbred	138	307,000.74**	3.49**	5.88**	3.01**	295.84**	80.45	1.05**	3.14**	0.08**	0.94**
Error	153	143,160.7	1.85	2.45	0.83	110.53	70.74	0.62	1.36	0.03	0.50
Repeatability (*R*)	—	0.41	0.64	0.55	0.33	0.59	0.58	0.17	0.02	0.18	0.43

*, **Significant at .05 and .01 probability levels, respectively.

Abbreviations: ASI, anthesis–silking interval; DA, days to 50% anthesis; DS, days to 50% silking; EASP, ear aspect (1–9); EHT, ear height (cm); Env, environment; EPP, ears per plant; GY, grain yield (kg/ha); PASP, plant aspect (1–9); PLHT, plant height (cm); STGR, stay‐green characteristic (1–9).

**Table 2:** Mean squares and heritability estimates of 70 early maturing provitamin A‐quality protein maize inbred lines evaluated under optimal conditions at Ile‐Ife, Ikenne and Mokwa in the 2016 and 2017 growing seasons and across all three research conditions

Source	*df*	GY	DA	DS	ASI	PLHT	EHT	PASP	EASP	EPP	DF	STGR
Optimal conditions												
Env	2	134,313,818.6**	101.29**	362.52**	81.52**	45,577.87**	20,840.08**	118.98**	173.25**	7.55**	—	—
Rep(Env)	3	21,773.4	0.54	1.57	0.93	694.99**	420.69**	10.25**	9.62**	0.13**	—	—
Block(Env × Rep)	54	103,579.7	5.55**	7.96**	1.00**	311.75**	118.27**	1.33*	1.31	0.05*	—	—
Inbred	69	768,236.7**	14.95**	18.25**	1.43**	633.41**	220.14**	1.68**	2.53**	0.06**	—	—
Env × Inbred	138	490,480.8**	2.18	3.05*	1.02**	141.65	85.31*	1.12*	1.22	0.04*	—	—
Error	153	73,194.1	1.86	2.26	0.58	111.5689	60.91	0.83	1.03	0.03	—	—
Repeatability (*R*)	—	0.58	0.87	0.86	0.54	0.81	0.65	0.55	0.54	0.52	—	—
Across research conditions												
Env	7	82,217,326.1**	191.49**	1,051.91**	404.54**	64,747.46**	10,398.83**	98.33**	221.51**	9.13**	4	126.47**
Rcond	2	119,609,298.8**	405.11**	2,800.80**	1,228.83**	164,727.14**	10,498.15**	167.84**	577.05**	20.05**	1	126.19**
Rep(Env)	8	263,665.7**	2.16	2.96	3.26*	773.13**	529.22**	5.13**	5.41**	0.08**	5	2.28**
Block(Env × Rep)	144	168,729.5**	7.24**	9.33**	1.7	303.55**	112.11**	1.58**	2.37**	0.05**	90	1.11**
Inbred	69	661,182.5**	31.09**	37.33**	5.02**	1,477.70**	432.19**	1.95**	4.30**	0.08**	69	1.50**
Rcond × Inbred	138	399,198.2	4.94	7.5	3.64**	209.15	72.7	1.46	2.85**	0.08	69	2.38**
Env × Inbred	483	346,564.9**	3.54	5.66**	2.96**	195.14**	75.56*	1.12**	2.24**	0.06**	276	1.28**
Error	408	89,760.6	3.17	4.37	1.45	106.54	62.9	0.7	1.16	0.03	255	0.56
Repeatability (R)	—	0.46	0.89	0.86	0.43	0.87	0.84	0.43	0.49	0.54	—	0.56

*, **Significant at .05 and .01 probability levels, respectively.

Abbreviations: ASI, anthesis–silking interval; DA, days to 50% anthesis; DS, days to 50% silking; EASP, ear aspect (1–9); EHT, ear height (cm); Env, environment; EPP, ears per plant; GY, grain yield (kg/ha); PASP, plant aspect (1–9); PLHT, plant height (cm); Rcond, research condition; STGR = stay‐green characteristic (1–9).

Mean squares for all carotenoids revealed significant (*p* ≤ .05) variation among the inbred lines assayed (Table 3) across two locations (Ibadan and Mokwa). Location mean squares were highly significant (*p* < .01) for all carotenoids except for β‐cryptoxanthin. Inbred × location interaction mean squares were significant (*p* < .01) for the measured carotenoids with the exception of β‐cryptoxanthin and α‐carotene. Compared to the error term, larger mean squares were observed for location, inbred lines and their interaction for the measured carotenoids. This was an indication of high variation between locations and among inbred lines across locations. Generally, lower coefficients of variation (CVs; <20%) were recorded for the carotenoids and DS.

**Table 3:** Mean squares of carotenoids of the early maturing provitamin A‐quality protein maize inbred lines across two well‐watered conditions in Nigeria, 2018

Source	*df*	Carotenoids (μg/g dry weight)							
		Lutein	Zeax	β‐cryp	α‐Carotene	β‐Carotene	PVA	Tcaro	DS
Location	1	1,217.97**	352.26**	0.0016	83.54**	12.90**	66.29**	14,499.94**	3.21
Rep (location)	2	4.84	14.86*	5.00**	4.68*	1.55**	0.48	27.93	2.19
Inbred	69	42.42**	27.97*	10.65**	1.62*	7.39**	22.35**	112.82**	14.53**
Inbred × location	69	42.02**	18.02**	1.79	0.58	3.71**	6.34**	66.26**	4.13
Error	138	4.78	5.95	0.29	0.19	0.07	0.20	10.85	3.74
CV (%)	—	23.48	16.19	18.78	21.27	5.41	6.00	13.12	3.48
Repeatability (%)		1.00	35.58	83.48	63.95	49.71	71.62	41.27	83.29

*, **Significant at *p* < .05 and .01, respectively.

Abbreviations: DS, days to 50% silk emergence, PVA, provitamin A; Tcaro, total carotenoids; Zeax, zeaxanthin; β‐cryp, β‐cryptoxanthin;

### 3.2 Mean responses of inbreds for agronomic traits and provitamin A carotenoids

Ranking of the lines was based on the multiple trait base index across environments (Table 4). The inbreds recorded greater yield reductions under drought relative to those under low‐N and across the two stresses. The analysis of data across stress environments generally revealed that the inbreds that had increased ASI, higher STGR and high percentage GY reduction also had lower GY and negative selection indices. Thirty‐three inbred lines showed tolerance across stress environments based on the multiple trait base index with performance under optimal conditions serving as a check.

**Table 4:** Performance of early provitamin A‐quality protein maize inbreds (best 15 and worst 10) plus six checks under drought, low‐N, optimal and across environments

Inbred line	Grain yield (kg/ha)				Yield reduction (%)			ASI			STGR		M.I across
	Drought	Low‐N	Optimal	Across Env	Drought	Low‐N	Across stress	Drought	Low‐N	Optimal	Drought	Low‐N	Stress
TZEIORQ 12	307.29	1,651.55	1,882	1,280.34	83.67	12.25	39.99	3.71	0.02	0.59	4.44	3.48	9.90
TZEIORQ 48	321.02	1,617.67	1,773	1,237.36	81.90	8.78	36.88	3.64	0.29	0.56	4.69	3.94	8.26
TZEIORQ 6	701.74	994.21	1,180	958.51	40.51	15.71	28.11	3.75	−0.01	0.31	3.68	4.16	8.26
TZEIORQ 8	895.12	1,051.06	1,194	1,046.57	25.00	11.94	18.47	2.65	0.96	1.03	3.25	4.93	8.01
TZEIORQ 37	462.79	1,329.34	1,803	1,198.38	74.33	26.27	50.30	3.97	0.73	0.73	3.36	3.32	7.97
TZEIORQ 27	701.48	1,320.87	1,900	1,307.35	63.07	30.47	46.77	3.91	0.27	0.40	4.89	3.08	7.50
TZEIORQ 5	518.62	1,023.99	1,194	912.10	56.55	14.22	35.38	1.69	0.17	0.39	3.56	4.73	7.03
TZEIORQ 9	523.25	979.67	1765	1,089.33	70.36	44.50	57.43	3.22	1.19	0.64	4.10	4.26	6.20
TZEIORQ 28	567.56	1,297.60	2,075	1,313.43	72.65	37.47	55.06	3.57	0.92	0.12	4.22	4.41	5.70
TZEIORQ 17	270.38	1,175.9	1,351	932.35	79.98	12.95	46.46	3.30	0.52	0.24	4.61	3.34	4.79
TZEIORQ 40	227.28	1,292.32	1,457	992.10	84.40	11.28	47.84	3.23	0.66	0.7	4.70	3.78	4.69
TZEIORQ 26	342.42	1,096.03	1,310	916.11	73.86	16.33	45.09	3.09	0.06	0.37	3.70	4.75	4.65
TZEIORQ 60	283.41	917.45	1,149	783.23	75.33	20.14	47.74	4.19	1.01	1.23	2.94	2.82	3.47
TZEIORQ 65	418.76	1,160.89	2,034	1,204.48	79.41	42.92	61.16	3.36	0.48	0.96	4.21	3.79	1.87
TZEIORQ 24	399.10	401.64	519	439.75	23.03	22.54	22.78	3.57	−0.26	0.15	3.87	4.77	1.55
TZEIORQ 29	297.27	857.81	1,139	764.66	73.90	24.68	49.29	5.78	0.90	1.23	4.27	3.34	−0.20
TZEIORQ 59	147.54	769.54	2,086	1,001.16	92.93	63.12	78.02	4.25	0.55	1.50	4.56	2.90	−0.67
TZEIORQ 11	122.10	844.8	2,189	1,051.84	94.42	61.40	77.91	4.57	1.50	0.10	5.62	3.96	−1.74
TZEIORQ 20	364.47	571.58	1,055	663.78	65.46	45.84	55.65	3.85	0.28	1.16	4.14	4.63	−3.59
TZEIORQ 13	191.53	1,138.79	2,171	1,167.03	91.18	47.54	69.36	4.67	2.43	0.07	6.25	4.03	−3.85
TZEIORQ 55	272.98	1,227.95	1,427	976.11	80.88	13.97	47.42	5.42	0.01	1.46	4.78	4.23	−4.45
TZEIORQ 2	112.67	606.95	1,462	699.25	92.29	58.49	75.39	3.27	1.24	1.02	4.54	3.83	−5.52
TZEIORQ 52	119.43	599.08	1,548	755.48	92.28	61.30	76.79	4.29	0.69	0.56	4.97	4.46	−7.26
TZEIORQ 46	106.55	458.35	1,396	653.74	92.37	67.17	79.77	4.72	2.03	0.54	6.65	5.13	−10.89
TZEIORQ 41	100.00	842.94	1,191	711.14	91.60	29.19	60.40	6.55	2.56	0.79	6.07	4.31	−11.82
C1‐TZEQI 85	104.62	297.96	1,610	670.77	93.50	81.49	87.50	6.14	3.34	1.56	5.15	3.83	−13.22
C2‐TZEQI 91	144.89	986.91	1,685	938.78	91.40	41.41	66.41	5.60	2.22	0.05	5.45	4.05	−7.49
C3‐TZEQI 74	123.98	298.79	760	394.18	83.68	60.67	72.18	4.05	5.21	1.21	4.26	4.00	−13.26
C4‐TZEQI 82	109.31	853.60	1,342	768.34	91.86	36.40	64.13	6.15	2.26	1.94	3.54	3.86	−5.21
C5‐TZEI 129	336.09	1,273.23	1,843	1,150.85	81.77	30.92	56.34	4.47	0.42	0.63	4.59	3.22	4.57
C6‐TZEI 24	349.98	1,437.46	1,920	1,235.92	81.77	25.14	53.46	6.60	0.55	0.33	4.88	2.86	5.17
Mean	321	980	1,529	942	76.62	34.73	55.68	4.27	1.07	0.73	4.51	3.94	
SED	177.04	344.86	457.65	225.83	—	—	—	1.63	1.06	0.61	0.68	0.60	

Abbreviations: ASI, anthesis–silking interval; C1 to C6, checks 1 to 6, respectively; Env, environment; M. I, multiple trait base index; STGR, stay‐green characteristics.

Although kernels were sampled under well‐watered conditions for both locations, mean concentrations of PVA and the component carotenoids were consistently higher for the results of Ibadan compared to the Mokwa site (Table 5). However, the two‐tailed independent samples *t* test revealed that only the PVA and β‐carotene concentrations were significantly higher. Larger effect size of 1.21 was estimated for the significant difference observed for β‐carotene concentrations measured for the two locations. Conversely, very small effect size of 0.09 was found for the significant difference in PVA concentrations between the two locations. Estimated PVA contents of selected inbred lines across the two locations varied from 4.83 μg/g for TZEIORQ 48 to 14.62 μg/g for TZEIORQ 55 with a mean of 7.38 μg/g (Table S1). Zeaxanthin (44%) and lutein (27%) were the most predominant carotenoids vis‐à‐vis the β‐cryptoxanthin (8%) and α‐carotene (6%) which had lower values. Relative to the contents of the other carotenoids, lower levels of α‐carotene were measured for most of the inbred lines under each and across locations.

**Table 5:** Mean comparison of provitamin A and the component carotenoids of selected early maturing PVA‐QPM inbred lines under two well‐watered conditions in Ibadan and Mokwa in Nigeria, 2018

Inbred lines	PVA (µg/g)		β‐cryp (µg/g)		α‐carotene (µg/g)		β‐carotene (µg/g)	
	Ibadan	Mokwa	Ibadan	Mokwa	Ibadan	Mokwa	Ibadan	Mokwa
TZEIORQ 55	15.38	13.85	6.88	6.71	3.74	3.11	11.23	7.39
TZEIORQ 29	11.80	11.31	5.81	5.57	3.10	3.28	7.69	6.54
TZEIORQ 20	9.84	8.36	3.79	4.12	2.99	2.02	5.78	5.95
TZEIORQ 42	10.54	6.82	4.26	4.25	2.76	2.45	5.84	4.64
TZEIORQ 13	9.27	6.55	4.10	3.43	2.14	2.72	5.73	3.92
TZEIORQ 24	9.15	6.38	3.49	3.97	3.31	1.56	5.31	4.17
TZEIORQ 59	7.19	8.01	3.69	3.45	2.38	2.20	5.84	3.48
TZEIORQ 40	8.98	5.98	3.23	3.83	2.30	2.34	4.77	4.35
TZEIORQ 7	8.66	5.62	3.21	3.05	2.70	1.58	5.75	3.25
TZEIORQ 6	8.68	5.55	1.63	1.22	1.48	1.44	6.93	4.44
TZEIORQ 26	9.06	5.12	3.11	2.77	1.58	1.79	6.13	3.40
TZEIORQ 5	8.10	4.31	1.76	1.51	1.89	1.35	5.68	3.45
TZEIORQ 43	6.98	5.11	2.59	2.04	1.51	2.05	4.79	3.19
TZEIORQ 45	6.41	5.10	1.25	1.29	1.22	1.39	5.13	3.91
TZEIORQ 23	7.14	3.89	2.07	1.78	2.37	1.40	4.20	3.02
TZEIORQ 44	6.65	4.25	1.31	1.02	1.06	2.01	4.31	3.80
TZEIORQ 2	6.27	4.34	1.20	1.30	1.06	1.50	4.99	3.12
TZEIORQ 47	6.85	3.65	1.20	1.12	1.31	1.05	5.55	2.58
TZEIORQ 48	5.75	3.92	1.05	1.20	2.08	1.04	4.56	2.37
SED	0.66	0.88	0.81	0.66	0.65	0.29	0.39	0.33
Mean difference	2.35		0.11		0.25		1.75	
*t*‐Value	2.92**		0.20		1.05		3.73**	
Cohen's *d*	0.09		—		—		1.21	

**Significant at .01 probability level.

Abbreviations: β‐cryp, β‐cryptoxanthin; PVA, provitamin A.

### 3.3 Identification of inbred lines carrying favourable alleles of LcyE and crtRB1 genes

**Figure 1:**
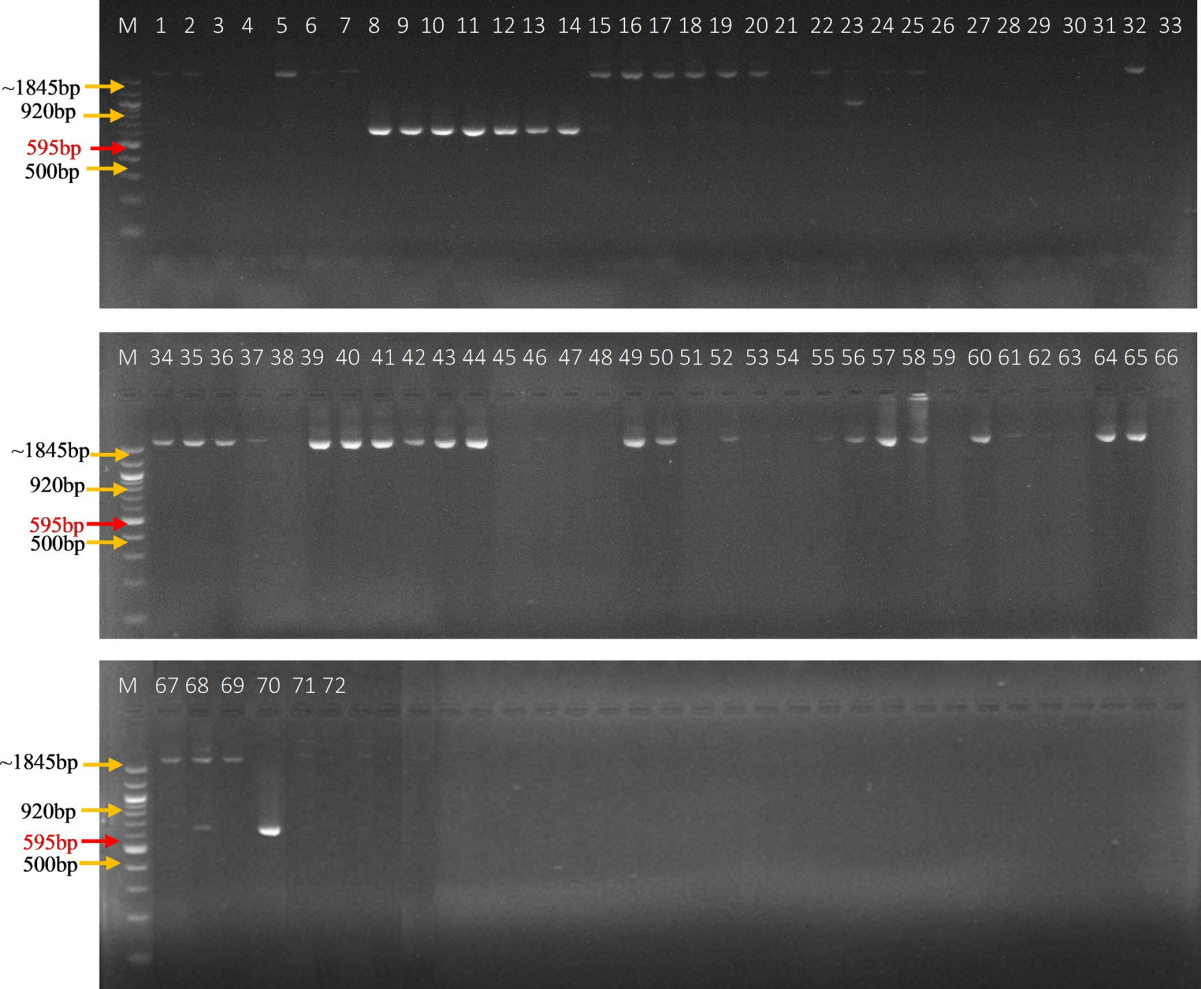
PCR amplification of selected early maturing provitamin A‐quality protein maize inbred lines using crtRB1_3TE_F1/RIC primer resolved on 2% agarose gel. *M* = 100 bp DNA ladder marker; favourable allele = 595 bp; unfavourable allele = 1,845 bp; lanes 1–70 = inbred lines with entry number as labelled; lanes 71–72 = no template controls (NTCs)

**Figure 2:**
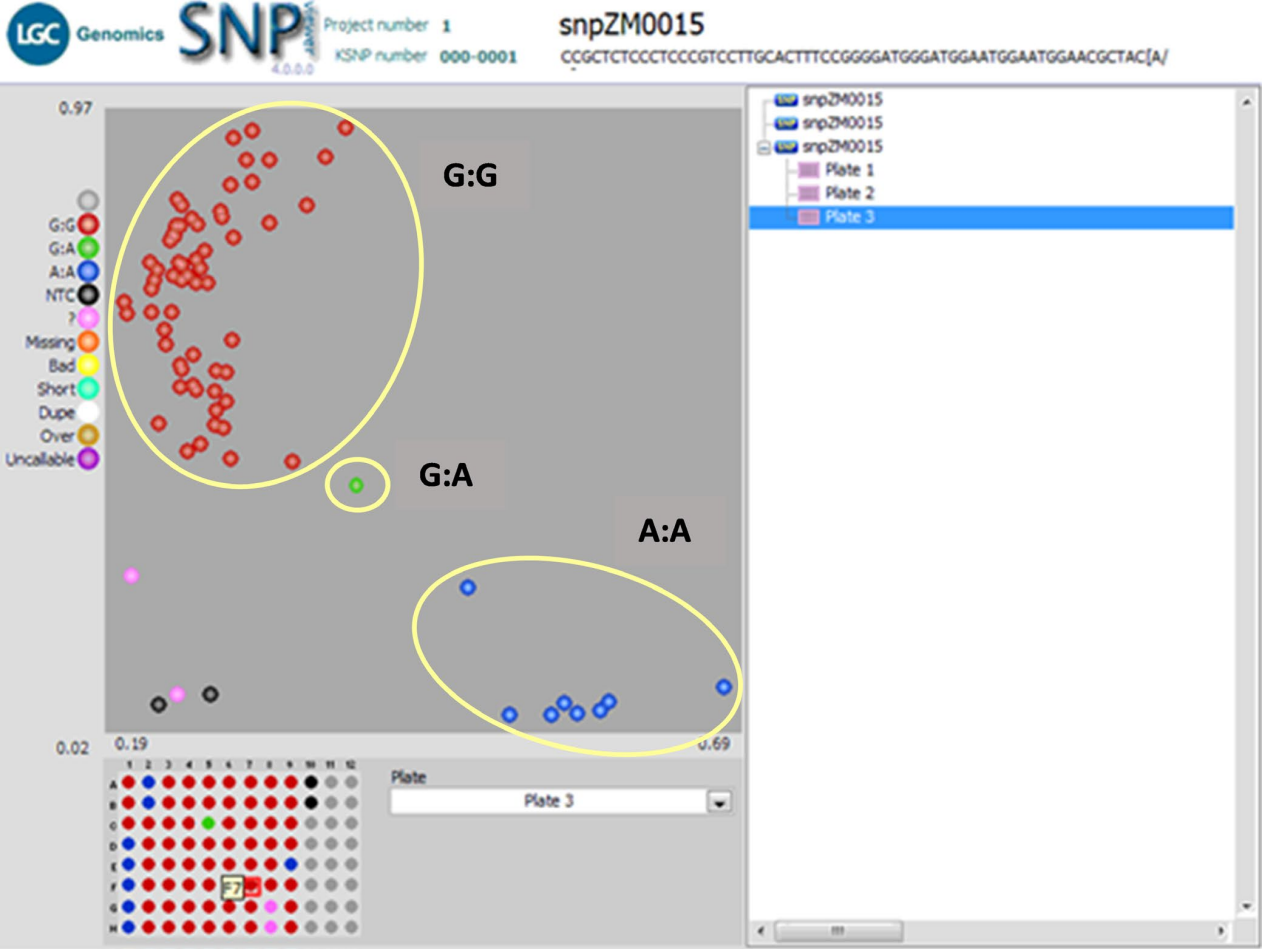
SNPviewer Image of 70 early maturing provitamin A‐quality protein maize inbred lines genotyped using KASP snpZM0015. A:A (blue) = inbreds with favourable allele; G:G (red) = inbreds with unfavourable allele; G:A (green) = heterozygous for the allele; (pink) = inbreds that did not amplify; NTC (black) = no template controls; snpZM0015 = KASP SNP targeting the crtRB1 gene

Among the 3 PCR‐based functional markers used, the crtRB1_3′TE_T_F1/R1C showed clear amplification with the expected band size (595 bp, labelled in red) for nine (~13%) out of the 70 inbred lines tested (Figure 1). The eight inbred lines carrying the favourable gene (*crtRB1*) were entries 8 to 14, and 70 representing inbred lines TZEIORQ 10, TZEIORQ 12, TZEIORQ 13, TZEIORQ 14, TZEIORQ 15, TZEIORQ 16, TZEIORQ 17, TZEIORQ 20 and TZEIORQ 55 in that order. TZEIORQ 55 had the highest PVA level of 14.62 μg/g (entry 68) and was identified as heterozygous for the crtRB1‐3′TE alleles. The inbreds identified to harbour the favourable alleles of *crtRB1* generally had moderate levels of PVA ranging from 6.01 μg/g for TZEIORQ 10 to 14.62 μg/g for TZEIORQ 55 (Table 6). Among the three primers used (*crtRB1‐3′TE*, *crtRB1‐5′TE* and *LcyE‐5′TE*), only *crtRB1‐3′TE* amplified with the band size of 595 bp across the entire panel of the inbred lines. The KASP SNP (snpZM0015) effectively distinguished between favourable and unfavourable alleles for the *crtRB1* gene in the 70 inbreds (Figure 2). All the eight inbreds that were amplified for the *crtRB1‐3′TE* allele using the gel‐based *crtRB1‐3′TE* marker were also identified by the KASP SNP, snpZM0015 for the same gene (A:A [blue] = lines with favourable allele). Thus, the KASP SNP targeted and selected the *crtRB1‐3′TE* polymorphism. Correspondingly, snpZM0015 identified TZEIORQ 55 to harbour the heterozygous alleles (G:A [green] = heterozygous) as revealed by the PCR‐gel‐based *crtRB1‐3′TE* marker. Fifty‐nine inbreds had the unfavourable allele (G:G [red] = inbred lines with the unfavourable allele), while no amplification was observed for two of the inbred lines (? [pink] = inbreds that did not amplify). The 2 non‐template controls [NTC (black) = no template controls] effectively checked the amplification and efficiency of the KASP SNP (snpZM0015) by clustering together away from the inbred samples (Figure 2).

**Table 6:** List of inbred lines carrying the functional gene (crtRB1)

Entry	Inbred line	Provitamin A (µg/g DW)
8	TZEIORQ 10	6.01
9	TZEIORQ 12	6.68
10	TZEIORQ 13	7.09
11	TZEIORQ 14	6.09
12	TZEIORQ 15	6.95
13	TZEIORQ 16	7.91
14	TZEIORQ 17	8.05
70	TZEIORQ 20	6.16
68	TZEIORQ 55	14.62

## 4 DISCUSSION

The significant variability among G for grain yield and most of the traits measured under each and across test environments implied the existence of genetic variability in the early maturing PVA‐QPM inbreds. The significance of E and GEI observed for GY and several other traits under each and across environments suggested the inconsistent rankings of the traits measured in varying environments and that inbred evaluations in more environments were necessary to identify outstanding cultivars under drought (Badu‐Apraku et al., 2011; Edmeades, 2013) and under low‐N (Meseka, Menkir, Ibrahim, & Ajala, 2006). The high repeatability (*R*) values estimated for PASP, EASP, EPP and STGR compared with that for GY under drought indicated that selecting for the yield related traits would be effective to complement GY in the identification of drought tolerant inbred lines (Bänziger, Edmeades, Beck, & Bellon, 2000). The moderately high R values estimated for DA, DS, PLHT and EHT under low‐N indicated that early generation testing of the inbred lines using these traits under low‐N conditions would be successful (Badu‐Apraku et al., 2013; Bänziger, Edmeades, & Lafitte, 1999).

The wide range of PVA values obtained across the locations indicated the existence of significant variation for the PVA carotenoids in the set of inbred lines used (Harjes et al., 2008; Mishra & Singh, 2010) and that each location revealed different genetic variations among the inbred lines. This suggested that G × E was significant for the measured carotenoids as evident in the significant mean squares of inbred × location interaction observed. Larger location mean squares compared to the corresponding error mean squares observed for the measured carotenoids suggested that although well‐watered conditions were ensured at both locations, other environmental differences resulting from edaphic and climatic factors regulated the accumulation of the different carotenoids. The two environments were therefore discriminating for the measured carotenoids. This finding agreed with the results of other workers who found significant but small genotype × environment interaction for PVA concentration in tropical maize (Egesel et al., 2003; Menkir et al., 2008; Menkir & Maxiya‐Dixon, 2004) and in temperate maize (Quakenbush, Firch, Brunson, & House, 1966). Significant genotype × environment interactions for PVA could result from differences in maturity periods (Cabrera‐Soto, Pixley, Rosales‐Nolasco, Galicia‐Flores, & Palacios‐Rojas, 2018). However, the inbred lines belonged to the early maturity (90–95 days) group and therefore the observed differences were not due to differences in maturity of the lines but could be attributed to the differences in the environmental conditions in Ibadan (forest‐savanna transitional zone) and Mokwa (southern Guinea savanna agro‐ecological zone). However, the results were contrary to the findings of other workers (Muzhingi et al., 2016; Suwarno, Pixley, Palacios‐Rojas, Kaeppler, & Babu, 2014) who observed non‐significant genotype × environment interaction effects for PVA concentration. The authors concluded that although environmental effect is a challenge in developing high PVA maize, the lack of genotype × environment interaction would aid selection for broad adaptation. The lower CVs observed for the traits across locations may have resulted in the consistency of mean carotenoids recorded for the two locations. Medium and moderately high *R* values were recorded for β‐carotene (50%) and α‐carotene (64%), while high *R* estimates were observed for β‐cryptoxanthin (84%). This might have resulted in the high *R* estimate of 72% for PVA. The practical implication of these results is that PVA concentrations of this set of inbred lines can be consistently reproduced from one planting season to the other. The high *R* estimate of 83% recorded for days to 50% silk emergence substantiated the classification of the inbred lines into the same (early) maturity group.

Forty‐seven per cent of the inbreds possessing drought and low‐N tolerance signalled the common adaptive mechanisms involved in the tolerance to the two stresses and that selection under drought could result in improved low‐N tolerance (Badu‐Apraku, Akinwale, Franco, & Oyekunle, 2012; Bänziger et al., 1999; Meseka et al., 2006). However, the greater yield reduction found under drought relative to that under low‐N and across the two stresses suggested that the drought conditions were more severe than low‐N and that with limited resources, selection for drought tolerance should be prioritized over low‐N. The inbreds identified would serve as an invaluable source of drought and low‐N tolerant genes for the development of superior hybrids and synthetics (Ifie, Badu‐Apraku, Gracen, & Danquah, 2015).

Carotenoid concentrations in most orange maize grain are predominantly lutein and zeaxanthin with lower levels for β‐cryptoxanthin and α‐carotene (USDA National Nutrient Database, ndb.nal.usda.gov). Results of this study corroborated this general trend. Beta‐carotene had the highest levels with respect to the PVA carotenoids. For the PVA levels of specific inbred lines, only TZEIORQ 55 recorded a value comparable to the target of 15 μg/g DW set by HarvestPlus (Harjes et al., 2008; HarvestPlus, 2004; Ortiz‐Monasterio et al., 2007) suggesting the need to introgress favourable PVA alleles to improve the tropically adapted early PVA‐QPM inbred lines. This would also help to facilitate the accumulation of PVA carotenoids in hybrids developed from these inbreds to exceed the set target (15 μg/g dry weight). Exceeding the PVA set target is a practically feasible approach to maximize the benefits of consuming PVA maize because studies have revealed that the loss in PVA during storage, milling and preparation of different traditional food items could be up to 70% (De Moura, Miloff, & Boy, 2015; Mugode et al., 2014; Muzhingi et al., 2008; Pillay, Siwela, Derera, & Veldman, 2014) and the degree of loss widely varies among maize genotypes.

Four functional markers, *crtRB1‐5′TE, crtRB1‐3′TE, LcyE‐5′TE* and KASP snpZM0015, were used to validate the most favourable PVA genes, *crtRB1 and LcyE* (Azmach et al., 2013; Babu et al., 2013; Harjes et al., 2008; Yan et al., 2010) for increased β‐carotene levels in a panel of 70 early maturing PVA‐QPM inbred lines. Functional markers are DNA markers capable of revealing polymorphisms in allele combinations which are responsible for the differences in phenotypes (Azmach et al., 2013; Babu et al., 2013; Harjes et al., 2008; Yan et al., 2010). From the results, the *crtRB1‐3*′*TE* was the most polymorphic marker and identified nine inbreds, TZEIORQ 10, TZEIORQ 12, TZEIORQ 13, TZEIORQ 14, TZEIORQ 15, TZEIORQ 16, TZEIORQ 17, TZEIORQ 20 and TZEIORQ 55, carrying the favourable alleles of the *crtRB1* gene. These inbreds could serve as donor parents of favourable alleles for the *crtRB1* gene. This result agreed with the findings of Yan et al. (2010), Babu et al. (2013) and Azmach et al. (2013) who validated the *crtRB1‐3′TE* functional marker in a PVA maize germplasm. However, with the exception of TZEIORQ 55, the PVA contents of the nine inbreds were moderate suggesting a situation of reduced gene expression (Hood, 2004; Mocellin & Provenzano, 2004) which could be due to gene silencing occurring during transcriptional or translational processes (Redberry, 2006). TZEIORQ 55 which recorded the highest PVA level of 14.62 μg/g DW across the two locations had the heterozygous form of the *crtRB1* gene. This was unexpected since all the inbred lines were at the S_8_. Perhaps one or a few of the 10 plants sampled for DNA for TZEIORQ 55 was an off‐type/ heterozygote. Therefore, tracking individual plant's DNA for this line may be imperative to identify the homozygotes. Nonetheless, further inbreeding may be required to fix the *crtRB1* gene of TZEIORQ 55.

The result of the KASP marker, snpZM0015, was in concordance with that of *crtRB1‐3′TE*, identifying the same nine inbreds to harbour the favourable alleles of the *crtRB1* favourable gene including TZEIORQ 55 as heterozygous for the *crtRB1* gene. It was striking to find the *crtRB1‐5′TE* and *LcyE‐5′TE* completely monomorphic for the alleles of the crtRB1 and LcyE genes in the entire set of inbred lines, yet some of the inbreds recorded moderate to high PVA contents. This result contradicts the findings of Yan et al. (2010), Harjes et al. (2008) and Azmach et al. (2013) who reported that *crtRB1‐5′TE* and *LcyE‐5′TE* are reliable markers for detecting the favourable alleles of the *crtRB1* and *LcyE* favourable genes. The result therefore suggested that genes other than the *crtRB1* and *LcyE* genes such as *PSY1* (Harjes et al., 2008) were responsible for the increased concentrations of the PVA carotenoids observed for some of the inbred lines. For instance, Owens et al. (2014) reported the *zep1* and *lut1* genes to be associated with increased levels of PVA carotenoids in the PVA biosynthetic pathway. Available knowledge of the favourable PVA genes and the phenotyping results of the carotenoids in this study implied that TZEIORQ 29 could serve as a donor parent of PVA favourable alleles other than those of *crtRB1* and *LcyE* genes.

In conclusion, TZEIORQ 55 and to some extent TZEIORQ 29 combined high PVA concentrations with drought and low‐N tolerance. The moderate range of PVA contents observed for most of the inbreds suggested the need to introgress favourable PVA alleles from temperate sources to improve the tropically adapted early PVA‐QPM inbred lines and to facilitate the accumulation of PVA in potential hybrids. The crtRB1‐3′TE primer and the KASP SNP (snpZM0015) consistently identified nine inbreds including TZEIORQ 55 to contain the favourable alleles of the crtRB1 gene. However, more comparative studies on the concordance between the crtRB1‐3′TE and snpZM0015 are required to improve the understanding between the two markers for efficient utilization in PVA maize development. Inbred × location interaction was significant indicating the need to evaluate genotypes across multiple locations when quantifying levels of PVA carotenoids in maize. The nine inbreds identified could therefore serve as donor parents of the favourable *crtRB1‐3′TE* allele, while TZEIORQ 29 could donate PVA favourable alleles other than those of *crtRB1* and *LcyE* genes for PVA maize breeding.

## CONFLICT OF INTEREST

The authors hereby declare that the study was carried out without any financial and/or commercial commitments that could result in a potential conflict of interest.

## AUTHOR CONTRIBUTIONS

E. Obeng‐Bio, B. Badu‐Apraku B.E. Ifie and A. Danquah designed this study. E. Obeng‐Bio and B. Badu‐Apraku performed the experiments. E. Obeng‐Bio and M.A Dadzie analysed the data. E. Obeng‐Bio and M.A Dadzie drafted the manuscript. B. Badu‐Apraku, E.T. Blay and B.E. Ifie revised the manuscript.
