# Supplementary material for: Phenotypic characterization and validation of provitamin A functional genes in early maturing provitamin A‐quality protein maize (Zea mays) inbred lines
Source: Plant Breed. 2019 Dec 20;139(3):575–88. doi: 10.1111/pbr.12798 (PMC7386915; doi:10.1111/pbr.12798)
Supplement: Supplementary file 1 [file PBR-139-575-s001.docx]

Supplementary Table 1. Carotenoid contents of selected early maturing provitamin A-quality protein maize inbred lines across two well-watered conditions (Ibadan and Mokwa) in Nigeria, 2018.

|  | **Carotenoids^§^ (μg g^-1^ dry weight)** | | | | | |  |  |
| --- | --- | --- | --- | --- | --- | --- | --- | --- |
| **Inbred lines** | **Lutein** | **Zeax** | **β-cryp** | **α-carotene** | **β-carotene** | ^⍕^**PVA** | **Tcaro** | **DS** |
| TZEIORQ 55 | 13.63 | 20.39 | 6.79 | 3.42 | 9.31 | 14.62 | 53.54 | 55.00 |
| TZEIORQ 29 | 16.53 | 10.76 | 5.69 | 3.19 | 7.12 | 11.56 | 43.29 | 61.00 |
| TZEIORQ 20 | 10.93 | 17.70 | 3.96 | 2.50 | 5.87 | 9.10 | 40.96 | 56.00 |
| TZEIORQ 42 | 7.18 | 17.06 | 4.26 | 2.61 | 5.24 | 8.68 | 36.35 | 55.00 |
| TZEIORQ 13 | 8.40 | 16.39 | 3.77 | 2.43 | 4.82 | 7.91 | 35.81 | 54.00 |
| TZEIORQ 24 | 8.11 | 16.37 | 3.74 | 2.44 | 4.71 | 7.80 | 35.37 | 55.00 |
| TZEIORQ 59 | 7.84 | 14.45 | 3.57 | 2.29 | 4.66 | 7.59 | 32.81 | 58.00 |
| TZEIORQ 40 | 8.46 | 15.77 | 3.53 | 2.31 | 4.56 | 7.48 | 34.63 | 54.00 |
| TZEIORQ 7 | 7.98 | 14.99 | 3.13 | 2.14 | 4.50 | 7.14 | 32.74 | 55.00 |
| TZEIORQ 6 | 3.67 | 8.20 | 1.43 | 1.46 | 5.68 | 7.12 | 20.44 | 55.00 |
| TZEIORQ 26 | 7.35 | 18.33 | 2.93 | 1.68 | 4.77 | 7.08 | 35.06 | 54.00 |
| TZEIORQ 5 | 15.45 | 15.16 | 1.65 | 1.62 | 4.57 | 6.20 | 38.45 | 55.00 |
| TZEIORQ 43 | 5.62 | 15.01 | 2.33 | 1.78 | 3.99 | 6.05 | 28.73 | 54.00 |
| TZEIORQ 45 | 8.54 | 14.21 | 1.27 | 1.30 | 4.51 | 5.79 | 29.83 | 55.00 |
| TZEIORQ 23 | 5.89 | 14.53 | 1.92 | 1.89 | 3.61 | 5.52 | 27.84 | 55.00 |
| TZEIORQ 44 | 11.24 | 13.67 | 1.16 | 1.53 | 4.10 | 5.45 | 31.70 | 53.00 |
| TZEIORQ 2 | 9.26 | 15.39 | 1.25 | 1.28 | 4.06 | 5.32 | 31.24 | 60.00 |
| TZEIORQ 47 | 9.97 | 14.29 | 1.16 | 1.18 | 4.08 | 5.25 | 30.68 | 54.00 |
| TZEIORQ 48 | 10.93 | 13.59 | 1.13 | 1.60 | 3.47 | 4.83 | 30.72 | 57.00 |
| SED | 3.11 | 2.37 | 0.67 | 0.42 | 0.86 | 1.13 | 4.07 | 1.33 |
| Min. | 3.67 | 8.20 | 1.13 | 1.18 | 3.47 | 4.83 | 15.47 | 53.00 |
| Max. | 16.53 | 20.39 | 6.79 | 3.42 | 9.31 | 14.42 | 39.87 | 61.00 |
| Mean | 9.31 | 15.07 | 2.88 | 2.03 | 4.93 | 7.38 | 25.11 | 55.00 |

Zeax= Zeaxanthin; β-cryp= β-cryptoxanthin; **^⍕^**PVA= provitamin A; Tcaro= Total carotenoids; DS= Days to 50% silk emergence across the two locations.
